# Characterization of Tetracalcium Phosphate/Monetite Biocement Modified by Magnesium Pyrophosphate

**DOI:** 10.3390/ma15072586

**Published:** 2022-03-31

**Authors:** Radoslava Stulajterova, Lubomir Medvecky, Maria Giretova, Tibor Sopcak, Lenka Luptakova, Radovan Bures, Eva Szekiova

**Affiliations:** 1 Division of Functional and Hybrid Systems, Institute of Materials Research of SAS, Watsonova 47, 040 01 Kosice, Slovakia; rstulajterova@saske.sk (R.S.); mgiretova@saske.sk (M.G.); tsopcak@saske.sk (T.S.); rbures@saske.sk (R.B.); 2 Department of Biology and Physiology, University of Veterinary Medicine and Pharmacy in Kosice, Komenskeho 73, 041 81 Kosice, Slovakia; lenka.luptakova@uvlf.sk; 3 Institute of Neurobiology of Biomedical Research Center of SAS, Soltesovej 4–6, 040 01 Kosice, Slovakia; szekiova@saske.sk

**Keywords:** calcium phosphate biocement, magnesium pyrophosphate, setting process, mesenchymal stem cells, gene expression

## Abstract

Magnesium pyrophosphate modified tetracalcium phosphate/monetite cement mixtures (MgTTCPM) were prepared by simple mechanical homogenization of compounds in a ball mill. The MgP_2_O_7_ was chosen due to the suitable setting properties of the final cements, in contrast to cements with the addition of amorphous (Ca, Mg) CO_3_ or newberite, which significantly extended the setting time even in small amounts (corresponding ~to 1 wt% of Mg in final cements). The results showed the gradual dissolution of the same amount of Mg_2_P_2_O_7_ phase, regardless of its content in the cement mixtures, and the refinement of formed HAP nanoparticles, which were joined into weakly and mutually bound spherical agglomerates. The compressive strength of composite cements was reduced to 14 MPa and the setting time was 5–10 min depending on the composition. Cytotoxicity of cements or their extracts was not detected and increased proliferative activity of mesenchymal stem cells with upregulation of osteopontin and osteonectin genes was verified in cells cultured for 7 and 15 days in cement extracts. The above facts, including insignificant changes in the pH of simulated body fluid solution and mechanical strength close to cancellous bone, indicate that MgTTCPM cement mixtures could be suitable biomaterials for use in the treatment of bone defects.

## 1. Introduction

The development of new bioactive calcium phosphate biocement systems (CPC) is a really interesting area in hard tissue reconstruction and regeneration medicine, filling bone defects and connecting various damaged bone structures. One of the frequently used CPC is the tetracalcium phosphate/monetite cement powder mixture due to its good mechanical properties, setting characteristics, and biological properties, as well as the final product of setting being calcium deficient nanohydroxyapatite [1]. Moreover, the monetite as an acidic calcium phosphate phase in the mixture has better osteoconductive and biodegradable properties than a hydroxyapatite (HAP) or brushite [2]. Mg^2+^ ions are most abundant in bone formation (~6 mol% substitution) during the initial stages of osteogenesis, tending to disappear when bone is mature [3]. Magnesium deficiency adversely affects all stages of skeletal metabolism, arresting bone growth, reducing osteoblastic and osteoclastic cell activity, osteopenia, and bone fragility [4]. In addition, magnesium deficiency has a direct effect on osteoporosis because it affects the formation of hydroxyapatite by bone cells, as well as parathyroid hormone secretion and activity [5]. The insignificant role of magnesium in osteogenic differentiation and proliferation of mesenchymal stem cells (MSCs) was found especially in more concentrated extracts of Mg-Ca alloy compared to pure Mg alloy, where the synergistic effect of both elements on cell activity was clearly demonstrated [6]. In the case of Mg-alloys, the corrosion resistance, biodegradation, adhesion, or proliferation of cells can be controlled by suitable coatings [7]. Despite increased 7 and 6.6 mM concentrations of Mg^2+^ and phosphate ions in the medium after partial dissolution of the compounds in cement mixture, no cytotoxicity, good proliferation, and increased ALP activity of osteoblasts cultured on hardened newberyite/brushite cement substrates were found [8]. In addition, magnesium and pyrophosphate ions significantly affected the nucleation and growth of HAP particles in solutions in a concentration manner [9,10].

MgO-based magnesium phosphate cements (MPC) are characterized by an exothermic reaction during cement hardening, which can be controlled by the addition of borax [11], and a strong basic pH in the range of 9–10, which can cause adverse and inflammatory reactions after in vivo application [11,12]. Strongly acidic farringtonite-based cement was prepared by chelating farringtonite with phytic acid [13]. Struvite cements used for biomedical applications had a high compressive strength of up to 50 MPa, a suitable setting time of 3–10 min, a high degree of solubility and promoted active bioresorption by osteoclasts [14], but the presence of ammonium ions in the cements could lead to undesirable ammonia release into human body. On the other hand, fast-setting types of cement limit the processing time for surgeons [15]. Struvite and newberyite cements have been shown to degrade more rapidly and transform into whitlockite or farringtonite after in vivo implantation, but a significant reduction in mechanical properties (>60%) has been found after 15 months of healing [16]. In composite cements based on calcium silicate or calcium sulphate/MPC, the reduction in thermal effect and pH during cement hardening has been investigated [17,18,19]. Magnesium indirectly affected bone mineralization through alkaline phosphatase (ALP) activation [20]. The development of cements containing magnesium and calcium phosphates (MCPC) has led to materials that combine the advantages of both components: high strength due to magnesium phosphate, lack of cytotoxicity and enhanced surface properties of the matrix due to the presence of calcium phosphates [21]. CPC/MPC cement composites had good bioactivity to form an apatite layer on the cement surface [22]. TTCP/monetite/struvite composite cement had high compressive strength (CS) and short setting time [23]. CPC/struvite composites have shown good biocompatibility and osteogenic potential in vivo [23,24]. Mg^2+^ ions released into the medium in vitro promoted the activity of osteoblasts [25] and osteoclasts [26]. Magnesium-potassium phosphate cement with 3 wt % by the addition of borax or 8 wt % by the addition of HAP reached CS~50 and 25 MPa, but a large amount of unreacted MgO was identified in the cement after hardening [27]. Unlike struvite cement, the newberyite/brushite cement, after setting with a powder cement mixture composed of farringtonite or Ca_3_Mg_3_(PO_4_)_4_ (stanfieldite) phase with 0.5 M citric acid, had wet CS up to 40 MPa and ST was reduced from 60 to 8 min as the result of refinement of cement particles by milling [8]. The stanfieldite and farringtonite cement mixtures partially transformed to struvite during hardening achieved only about 10 MPa to 6 MPa after 1 and 30 days setting in PBS, respectively, due to enhanced dissolution of cements with rise in porosity [15]. Note that all above reported CPC composites contained either a high portion of magnesium phosphate component or concentrated solutions of phosphates, citrates, and ammonium ions for improving CS and reduction in ST.

The aim of this paper was to characterize biocement tetracalcium phosphate/monetite powder mixture (TTCPM) modified by a small addition of Mg_2_P_2_O_7_ (MgTTCPM) which was not yet studied. The average content of Mg in bones does not exceed 1–2 wt% [28,29] and the composition of synthesized MgTTCPM powders was kept close to this level of Mg content. The various magnesium compounds, which differed by solubility, were tested in cement mixture and the final cement composition was optimized from the point of view of the setting process, compressive strength, and in vitro cellular response. The physico-chemical properties, setting process, microstructure, and phase evolution of MgTTCPM during setting were evaluated. Moreover, in vitro cytotoxicity testing of cement extracts and their effect on mesenchymal stem cell proliferation as well as osteogenic gene expression were analyzed.

## 2. Materials and Methods

### 2.1. Preparation of Cement Mixture and Samples for Evaluation

The CPC powder mixture was composed of TTCP and monetite (DCPA, calcium hydrogen phosphate anhydrous, Ph.Eur, Merck, Darmstadt, Germany) in equimolar ratio and was prepared according to a method in Ref. [30]. The chemical composition of MgTTCPM hardened cement was determined by the chemical analysis ICP (Horiba Activa, HORIBA Jobin Yvon Inc., Park Avenue, Edison, NJ, USA) after dissolution of cement in HNO_3_ (20%, analytical grade, Merck, Darmstadt, Germany). Magnesium pyrophosphate was obtained by annealing newberyite (MgHPO_4_·3H_2_O, Sigma Aldrich, Steinheim, Germany) at 850 or 1000 °C for 1 h. For selection of proper magnesium compound applicable in cement, setting time and resistance to wash out of TTCPM cements with addition of Mg_2_P_2_O_7_, (Ca, Mg) CO_3_ (amorphous phase synthesized fast precipitation from mixed solution of calcium and magnesium nitrates using sodium carbonate, following precipitate was filtered, washed with distilled water and ethanol, and dried at 100 °C/2 h) or newberyite was evaluated. Selected magnesium compounds for the optimization of cement composition were added in an amount so that the Mg content in the final cements was 1 wt%. The MgTTCPM cement powder mixtures were prepared by simple homogenization of TTCPM and the magnesium component in a planetary ball mill (RETSCH GmbH, Haan, Germany, 200 rpm for 1 h, ethanol).

### 2.2. Characterization Methods

The 2% NaH_2_PO_4_ (analytical grade, Sigma-Aldrich, Steinheim, Germany) solution was used as a liquid component. The powder to liquid (P/L) ratio was 2. The compressive strength (CS) was measured on a universal testing machine (LR5K Plus, Lloyd Instruments Ltd., West Sussex, UK) at crosshead speed of 1 mm/min (mean + standard deviation, *n* = 5). The cement pastes were packed in stainless cylindrical form (6 mm D × 12 mm H) and hardened in 100% humidity at 37 °C for 10 min. Next, the samples were soaked in simulate body fluid (SBF) at 37 °C for 1 week—the hardened cements were assigned as C1MP850 (1 wt% of Mg added in Mg_2_P_2_O_7_ form annealed at 850 °C) and C1MP1000 or C2MP1000 (1 or 2 wt% of Mg added in Mg_2_P_2_O_7_ form annealed at 1000 °C). The phase composition of samples was analyzed by X-ray diffraction analysis (Philips X PertPro, Malvern Panalytical B.V., Eindhoven, The Netherlands) using Cu Kα radiation, 50 mA, 2 Θ range 20–40°) and FTIR spectroscopy (Shimadzu, Kyoto, Japan, IRAffinity1, 400 mg KBr + 1 mg sample).

The microstructures of the cement fractured surfaces, after 7 days hardening in SBF, were observed by the field emission scanning electron microscopy (JEOL FE SEM JSM-7000F, Tokyo, Japan) after coating with carbon. The morphology of particles in the samples was observed using transmission electron microscopy (JEOL JEM 2100F, Tokyo, Japan).

The final setting times (ST) of the cement pastes were evaluated using the tip (1 mm diameter) of a Vicat needle with a 400 g load (according to ISO standard 1566), when it failed to make a perceptible circular indentation on the surface of the cement.

The amount of released calcium, phosphate, and magnesium ions from cements during soaking of cement pellets in SBF solution at 37 °C (400 mg/15 mL solution, polypropylene tube and shaken) were determined by ICP OES (Horiba ActivaHoriba Activa, HORIBA Jobin Yvon Inc., Park Avenue, Edison, NJ, USA) after filtration over the membrane filter (PVDF, 45 µm pore size, Millipore) at selected soaking times (0, 2, 4, 6, 24, 48, and 168 h).

### 2.3. Preparation of Mg Cement Extracts and In Vitro Cytotoxicity Testing of Extracts

For in vitro experiments (long-term cytotoxicity, ALP activity, gene expression, and Western blotting), the rat MSCs isolated from femur and tibia bone marrow were used and characterized [31].

For preparation of cement extracts, cement pastes were soaked in complete osteogenic differentiation culture medium (α-modification Eagle’s minimum essential medium, EMEM; Biosera, Marikina, Philippines), 10% FBS, osteogenic supplemented with 50 μg/mL of L-ascorbic acid, 50 nM dexamethasone, 10 mM β-glycerophosphate, and 1% penicillin, streptomycin, and amphotericin (all Sigma-Aldrich, Saint Louis, MO, USA) in an incubator at 37 °C for 24 h. A ratio of 0.2 g cement powder/mL of medium (ISO 10993-12:2012 [32], O-extracts) was used for 24 h in vitro cytotoxicity testing using pre-osteoblastic cell line MC3T3E1 Subclone 4 cells (ATCC CRL- 2593, Manassas, VA, USA). Extracts were sterilized by filtration through a membrane filter (0.2 μm pore size, Millipore, PVDF).

MC3T3E1 cell density in suspension with culture medium was adjusted to 1.0 × 10^5^ cells/mL in a vial. The 1.0 × 10^4^ of mouse preosteoblasts were suspended in 100 μL of medium (EMEM + 10% FBS, 1% antibiotic solution), seeded to wells of a 96-well microplate (cell grade Brand, adherent wells), cultured in an incubator for 24 h and after replacing the medium with 100 μL of O extracts, and cells were cultured for 24 h in incubator at 37 °C, 95% humidity, and 5% CO_2_. Then, the extracts were replaced with fresh culture medium and the in vitro cytotoxicity was evaluated (ISO 10993-5:2009 [33]) using the MTS proliferation test assay (cell titer 96 aqueous one solution cell proliferation assay, Promega Madison, WI, USA) and a UV–VIS spectrophotometer (Shimadzu, Kyoto, Japan).

For long-term cytotoxicity testing of cement extracts, a ratio of 0.1 g cement/mL medium was selected (M extracts). The cell density of resuspended rat MSCs passage 3 in culture medium was adjusted to 5.0 × 10^4^ cells/mL in a vial. and, 2.0 × 10^4^ of rat MSCs were added to 400 μL of EMEM + 10% FBS, 1% antibiotic solution and seeded into each well of a 48-well plate (TPP, Trasadingen, Switzerland). After cultivation for 24 h in an incubator of cells to a semi-confluent monolayer (at 37 °C, 95% humidity, and 5% CO_2_), the culture medium in the wells was replaced with 400 μL of M extracts. All cytotoxicity tests were carried out in triplicate, and cells cultured in extract-free complete culture medium were considered as the negative control. Extracts were changed every two days.

Mg cement samples (Ø 6 mm, 1 mm in thickness) were sterilized in an autoclave and placed in wells of 48–well culture plate and the MC3T3E1 cell suspension was adjusted at a density 5 × 10^4^. To each tested sample was 2 × 10^4^ cells in 400 μL of culture medium added. After 2 days of culturing, the density, distribution and morphology of the cells were assessed by live/dead fluorescent staining (fluorescein diacetate FDA/propidium iodide PI) by an inverted optical fluorescence microscope (Leica DM IL LED, Japan, blue filter).

The ALP activity of osteoblastic differentiated MSCs was determined in cell lysates (0.1% Triton X-100, 1 mM MgCl_2_ a 20 mM Tris) after freezing thawing and centrifugation at 10.000 RPM for 10 min. The solution of equal amounts of cell lysate and phosphatase substrate (p-nitrophenyl phosphate in dietanolamine buffer (0.5 mM MgCl_2_, pH = 9.8)) were incubated at 37 °C for 60 min after stopping reaction with 50 μL of 3 M NaOH. The ALP activity (in nanomoles of the p-nitrophenol produced per 1 min per microgram of proteins) was expressed by the amount of p-nitrophenol produced during the ALP enzyme catalysis of the p-nitrophenyl phosphate substrate, which was determined using the UV VIS spectrophotometer at 405 nm. The content of proteins in lysates was evaluated by Bradford’s method with Coomasie blue G250 as the complexing agent.

### 2.4. Gene Expression and SDS PAGE Analysis of Specific Markers in Differentiated Rat MSCs in Long-Term Culture

The gene expression was analyzed according to method as in Reference [30]. For the extraction of total RNA, 1 × 10^6^ cells were used. The quantification of genes in the cDNA samples was performed using primers for the following genes: B-actin rat, type I collagen rat, osteocalcin rat, osteopontin rat, osteonectin rat, and alkaline phosphatase rat (Table 1). cDNA for β actin was used as the endogenous control for calculating fold differences in the RNA levels of cells treated vs. not treated with cement extracts according to the 2^−∆∆CT^ method.

**Table 1:** Forward (F) and reverse (R) primers of genes used for RT-PCR experiments.

Genes	Primers (5′–3′)	References
B-actin rat	F: GTAGCCATCCAGGCTGTGTT R: CCCTCATAGATGGGCAGAGT	[34]
Osteocalcin rat	F: CCAGCTGACCTTCCTGCGCC R: CGGTGTGACTCGTGCAGCCA	[35]
Osteonectin rat	F: ACAGACAAGTCCCACACAGCAACT R: CCTGCTTGGACATGAAGGCTTTGT	[36]
Osteopontin rat	F: CCGATGAATCTGATGAGTCCTT R: TCCAGCTGACTTGACTCATGG	[37]
Alkaline phosphatase rat	F: GGAAGCTGCAGAAGAGATGG R: TGCACACCTTTTCAAACTCG	[37]
Osteonectin rat	F: AACCTGACTGACCCTTCCCTCT R: TCAATCCTGCCTCCTTCCACTA	[38]

The plate was sealed using an optical adhesive cover (Roche, Basel, Switzerland) and placed in a LightCycler 480 II real time PCR system machine (Roche, Switzerland). Real time PCR was performed under the following conditions: initial incubation at 95 °C for 10 min, amplification in 45 cycles at 95 °C for 15 s followed by 60 °C for 1 min. Amplification specificity was checked by generation of a melting curve.

SDS PAGE analysis were prepared from MSCs cultured in long-term extracts, washed with cold PBS, collected and lysed in denaturing cell extraction buffer (Invitrogen, Carlsbad, CA, USA, ThermoFisher, Waltham, MA, USA) supplemented with 1 mM PMSF (phenylmethylsulfonyl fluoride, Sigma-Aldrich, Steinheim, Gernmany) and SIGMAFAST protease inhibitors tablets (Sigma-Aldrich). Similar protein amounts (about 20 mg in 30 μL) were analyzed using SDS-PAGE with precast commercial 10% Bis-Tris mini gels (NuPAGE, Invitrogen, Cartsbad, CA, USA) with 1× MES buffer (NuPAGE Novex, Invitrogen) and Western blot analysis was carried out using transfer buffer (NuPAGE, Novex, USA) and the semi-dry method (FastBlot semi-dry blotter, Biometra, Germany) according to the method described in Reference [30]. Lanes were detected by chemiluminescence with Pierce ECL Plus Western Blotting Substrate (Pierce Biotechnology, Rockford, IL, USA) and were evaluated using ImageJ software.

## 3. Results

### 3.1. Compressive Strength and Setting Time of Cements

For selection of appropriate magnesium compounds for utilization in TTCPM cement system, the final setting times (ST) of cement mixtures were firstly analyzed. As it was stated, three different magnesium compounds-Mg_2_P_2_O_7_, (Ca, Mg) CO_3_, or newberyite–were analyzed from the point of view setting process of the powder cement mixtures with addition of 1 wt% of Mg in relation to chemical formulae of the given compound. The ST of C1MP850 cement was 10 ± 1 min contrary to ST of C1MP1000 or C2MP1000 equal to 5 ± 1 min. On the other hand, 1 wt% of Mg addition in the form of (Ca, Mg) CO_3_ or newberyite to the TTCPM mixture caused strong prolongation of the cement setting, and ST of cements was >45 min, which is unacceptable for medical use. For this reason, the Mg_2_P_2_O_7_ was selected as a magnesium cement additive in further study.

The compressive strength of C1MP cements and C2MP1000 were close to 15 ± 2 MPa and were reduced as compared to the CS of TTCPM cement equal ~39 ± 4 MPa.

The relative densities of hardened cements were close to 50.5% of theoretical HAP density and no statistically significant differences were found between samples.

### 3.2. Release of Ca^2+^, Mg^2+^, and Phosphate Ions from Cements to SBF and pH Measurement

**Figure 1:**
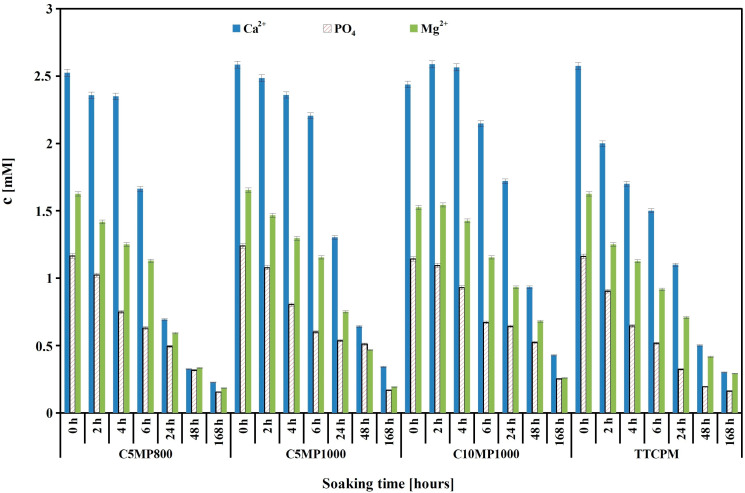
Release of calcium, magnesium, and phosphate ions during soaking of C1MP850, C1MP1000, C2MP1000, and TTCPM samples in SBF at 37 °C.

The changes in concentration of released Ca^2+^, Mg^2+^, and phosphate ions during soaking of cements in SBF at 37 °C are shown in Figure 1. It is clear from the comparison that the decrease in the concentrations of ions with soaking time were very similar, and absolute values of maxima and minima of concentrations were almost identical, regardless of the annealing temperature of magnesium pyrophosphate or its content in the cements. Note that despite the content of Mg_2_P_2_O_7_ in C2MP1000 being double that in C1MP1000 cement, a small rise in concentration of magnesium or phosphate ions was found during the first stage of setting (up to 4 h) only. In an addition, results from the analysis of solutions after the release of ions from the cements containing (Ca, Mg) CO_3_ or newberyite (the total content of these phases was almost double that of the content of Mg_2_P_2_O_7_ in cements because of they contain only ~13 wt% contrary to ~22 wt% of Mg and an amount of magnesium was kept ~1 wt%) after 2 h of soaking showed that concentrations of Ca^2+^, Mg^2+^, and phosphate ions were 3.9, 6.5, 1.05, 1.96, 3.93, and 4.75 mM for (Ca,Mg)CO_3_ or newberyite modified TTCPM cements, respectively, which were much higher in Mg^2+^ than that in TTCPM cements modified with Mg_2_P_2_O_7_. Only a small shift in pH of SBF to 7.5 was measured during soaking of all cements.

### 3.3. XRD and FTIR Analyses of Powder Mixtures and Cements

**Figure 2:**
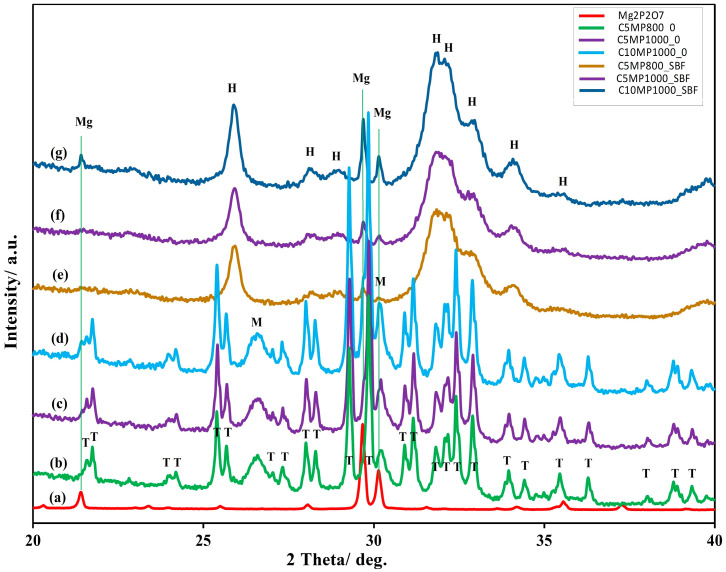
XRD patterns of origin cement powder mixtures and Mg_2_P_2_O_7_ before (Mg_2_P_2_O_7_ (**a**), C1MP850-0 (**b**), C1MP1000-0 (**c**), C2MP1000-0 (**d**)), and after 7 days soaking in SBF at 37 °C (C1MP850-SBF (**e**), C1MP1000-SBF (**f**), C2MP1000-SBF (**g**)). (Mg: Mg_2_P_2_O_7_ (JCPDS 32-0626), H: hydroxyapatite (PDF4 01-071-5048), M: monetite (JCPDS 09-0080), TTCP (JCPDS 25-1137)).

Figure 2 shows the XRD patterns of the starting cement powder mixtures and hardened samples after 7 days soaking in SBF (after 1 min crushing in agate mortar with a pestle). In the original MgTTCPM cement powders, the TTCP (JCPDS 25-1137), monetite (JCPDS 09-0080, reflections from (020), (−220), and (−112) planes at 2Θ ~ 26.51 and 30.21°) and Mg_2_P_2_O_7_ (JCPDS 32-0626) lines (Figure 2a–d) were found. After soaking for 7 days in SBF, the full transformation of starting calcium phosphate phases to nanocrystalline hydroxyapatite (PDF4 01-071-5048) was revealed. Moreover, the remains of Mg_2_P_2_O_7_ were identified in XRD patterns after hardening.

Vibrations from a symmetric stretching (962–941 cm^−1^) mode (ν_1_) of TTCP [39,40]; shoulders at 1134 and 900 cm^−1^ arising from ν_3_ stretching vibrations of P–O and P–O(H) monetite bonds and OH plane bending monetite vibrations at 1410 and 1351 cm^−1^ were found [41]. The characteristic vibrations of pyrophosphate group can be clearly distinguished in the spectrum of Mg_2_P_2_O_7_–bands at 737, 758, 972, and 992 cm^−1^, representing symmetric and antisymmetric P-O-P vibrational stretching mode, respectively; bands at 1209–1050 cm^−1^ P-O stretching vibrations from PO_3_ and the region of wavenumbers between 600–500 cm^−1^ is attributed to the bending mode of O–P–O bonds [42,43,44]. The comparison of the spectra shows that the Mg_2_P_2_O_7_ phase was visible in the spectra of the powder cement mixtures only through a peak at 736 cm^−1^ of lower intensity due to the reduced content of this phase.

**Figure 3:**
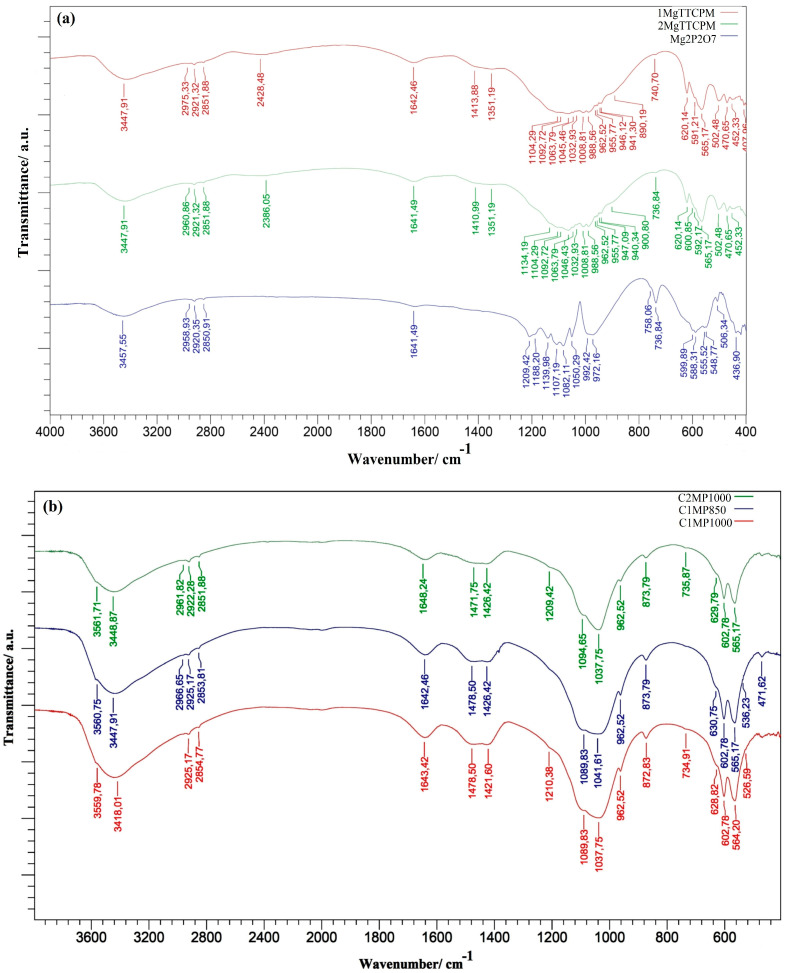
FTIR spectra of (**a**) 1 MgTTCPM, 2 MgTTCPM powder mixtures and Mg_2_P_2_O_7_; (**b**) FTIR spectra of C1MP850, C1MP1000, and C2MP1000 hardened cements after 7 days soaking in SBF.

The FTIR spectra of hardened cements after 7 days soaking in SBF demonstrate almost fully transformation of starting calcium phosphate phases to nanocrystalline hydroxyapatite (Figure 3b). In spectra, the stretching and librational modes of OH group in hydroxyapatite at 3560 cm^−1^ and 630 cm^−1^, respectively, are clearly visible. In addition, the stretching symmetric vibrations ν_1_ at 962 cm^−1^, O–P–O bending vibrations from triplicate degenerate bending mode (ν_4_) at 602 and 567 cm^−1^, doubly degenerated bending vibrations ν_2_ at 471 cm^−1^, and vibration from triple degenerated antisymmetric stretching mode ν_3_ at 1089 and 1037 cm^−1^ arise from the phosphate group of hydroxyapatite [45]. The presence of carbonates in hydroxyapatite clearly indicates bands at 1478, 1420, and 873 cm^−1^ which represent ν_3_ (asymmetric stretching) and ν_2_ (out-of-plane bending) vibrational modes of carbonate substituted for a phosphate group in B-type carbonated hydroxyapatite [45,46]. Besides, remains of pyrophosphates represented by very low intense peaks at 1210 and 735 cm^−1^ were identified in spectra which correspond with findings in XRD analysis. Note that broader low intense and poorly resolved peaks from vibrations of the hydroxyl group in hydroxyapatite are characteristic for nanocrystalline hydroxyapatite form.

### 3.4. SEM and TEM Analyses

The analysis of particle size distribution of Mg_2_P_2_O_7_ phase after milling showed monomodal curves regardless of annealing temperature with a small shift of d_50_ from 18.5 to 21.5 µm at 1000 °C due to the coarsening of particles at the higher temperature.

**Figure 4:**
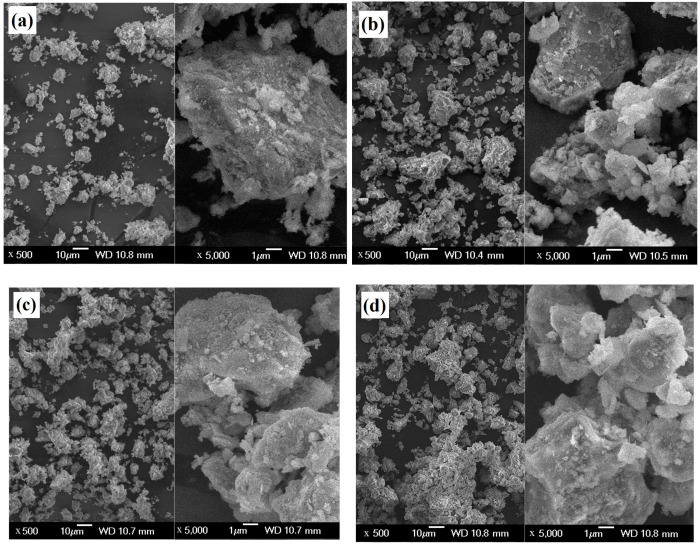
Morphology of TTCPM (**a**), 1 Mg TTCPM850 (**b**), 1 Mg TTCPM1000 (**c**), and 2 Mg TTCPM1000 (**d**) starting powder cement mixtures.

After mixing of this phase with TTCPM in a ball mill, the SEM micrographs showed refinement of particles because no bigger particles (>10 µm) of magnesium pyrophosphate were found in the powder mixtures, and the pyrophosphate phase was almost uniformly distributed in powders which verified EDX analysis in Figure 4b,d. Moreover, the coarser particle agglomerates (up to 10 µm in size) composed of fine separated nanoparticles which coated the surfaces of bigger TTCP particles were visible in the TTCPM powder (Figure 4a). In the case of MgTTCPM powder mixtures (Figure 4b–d), a stronger particle agglomeration with rise in pyrophosphate content was found with a maximum size of irregularly shaped agglomerates of ~20 µm and ~40 µm in 1 Mg TTCPM and 2 Mg TTCPM samples, respectively.

**Figure 5:**
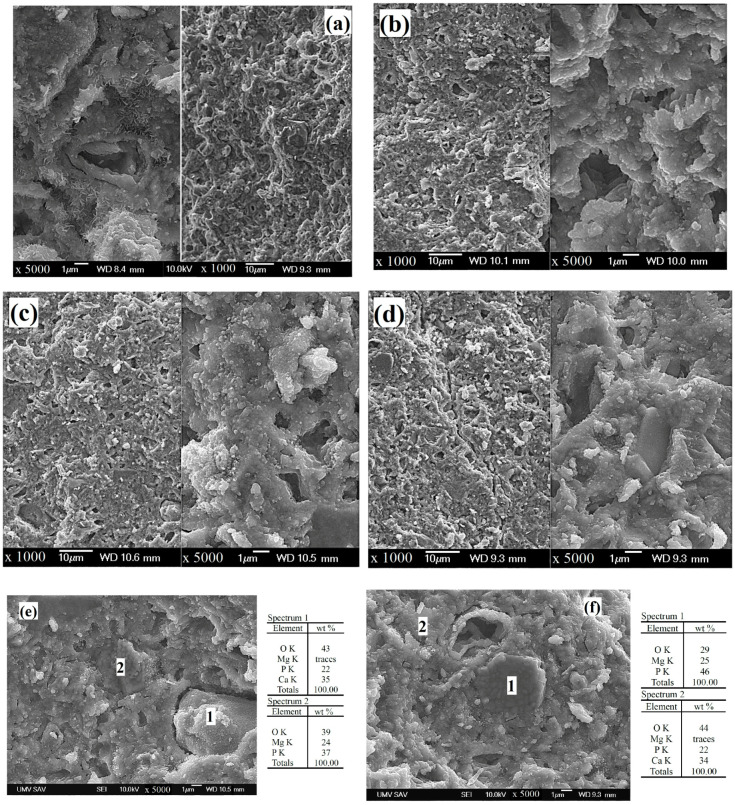
Microstructure of cements after 7 days of hardening in SBF at 37 °C: TTCPM (**a**), C1MP850 (**b**), C1MP1000 (**c**), C2MP1000 (**d**), EDX analysis of individual grains in C1MP1000 (**e**), and C2MP1000 (**f**) cements.

A high number of micropores with a size of 1–2 µm was observed in the microstructure of TTCPM cement (without magnesium) and larger micropores with a size of 5 µm had walls formed of a relatively compact hydroxyapatite layer with a thickness of about 1 µm composed of thin needle-like particles with a length of 100–200 nm (Figure 5a). These specific nanoparticles have strengthened the microstructure of the cement, especially at the grain boundaries, and have increased the fracture toughness of the hardened cement. No similar hydroxyapatite particle morphology was detected in the microstructures of the magnesium-containing cements.

In the microstructure of C1MP850 and C1MP1000 cements, a large proportion of irregularly shaped micropores with a size of 1–2 µm and a small pore fraction with a size of about 5–10 µm were identified after soaking in SBF. The microstructure was characterized by relatively compact globular agglomerates (size of 1–5 µm), which consisted of very fine spherical hydroxyapatite nanoparticles of submicrometric dimensions (up to 100 nm). The pulling out of these agglomerates from the microstructure is clearly visible on the fracture surface of the samples, which demonstrates a weaker interconnection between the individual agglomerates (Figure 5b,c). A similar microstructural character was observed on the fracture surface of C2MP1000 cement (Figure 5d), but in contrast to C1MP samples, a higher number of larger irregularly shaped pores with a size of about 10 µm was found, as well as coarser and smoother particles. Micrographs combined with EDX spectra (Figure 5e,f) revealed that the smoother, coarser particles were agglomerates of the remaining Mg_2_P_2_O_7_ phase containing globular particles (~1 μm in diameter, Figure 5e,f, spectra 2) sintered into a more compact grain during annealing of the newberyite precursor. Note that the pyrophosphate particles were tightly bound to the surrounding fine calcium phosphate matrix with no visible grain boundaries. In addition, the formation of fine hydroxyapatite nanoparticles on the surfaces of pyrophosphate particles was revealed in the figure. Similarly, coarse hydroxyapatite agglomerates (Figure 5e) derived from the transformation of TTCP particles were identified in the microstructure, but were very weakly bound to the cement matrix, as evidenced by their pull out from the microstructure and the existence of a separate hollow zone around the boundary.

**Figure 6:**
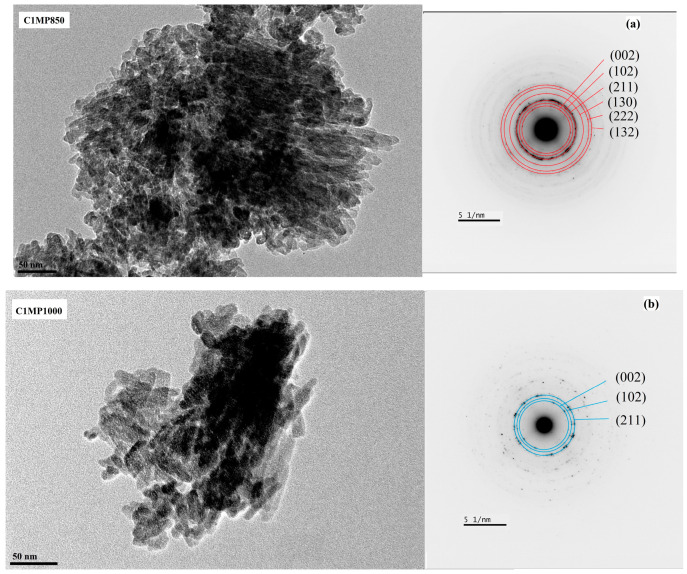

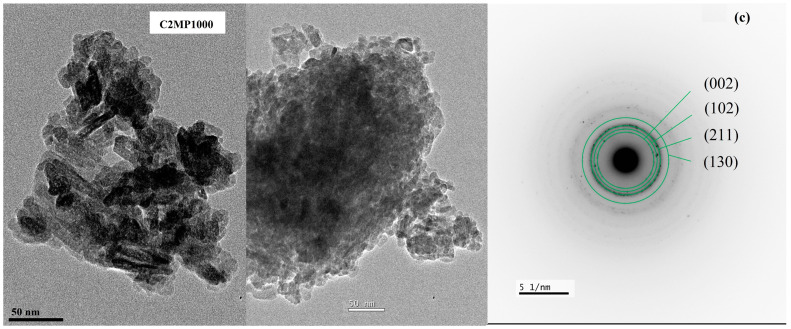
Morphology and SAED of hydroxyapatite nanoparticles in C1MP850 (**a**), C1MP1000 (**b**), C2MP1000 (**c**) observed using TEM.

**Figure 7:**
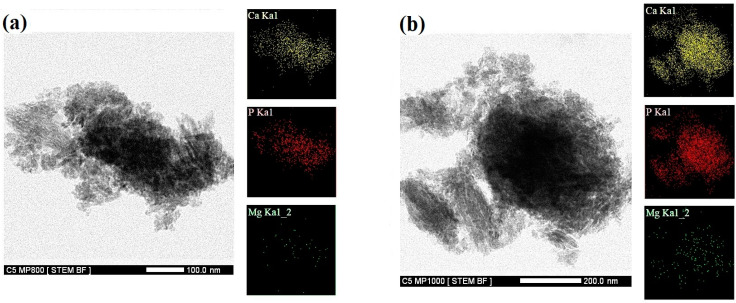
STEM/EDX analysis (mapping) of elements in C1MP850 (**a**), C1MP1000 (**b**).

For a more detailed analysis of the morphology of hydroxyapatite particles formed during the transformation of calcium phosphate phases in Mg TTCPM cements, a TEM analysis of the samples was carried out after hardening in SBF for 7 days and crushing. In the hydroxyapatite agglomerates (confirmed by selected area electron diffraction (SAED), PDF4 01-071-5048) of all cements, a high proportion of very fine spherical particles with a diameter of 5 to 20 nm combined with rod-shaped particles with a thickness of 20 nm and a thickness of up to 50 nm in length was identified (Figure 6). As can be seen in the EDX analysis of small agglomerates of hydroxyapatite nanoparticles on STEM images of C1MP850 and C1MP1000 cements (Figure 7a,b), magnesium was uniformly distributed in agglomerates that show homogeneous co-precipitation of calcium and magnesium ions to the hydroxyapatite phase after cement conversion.

### 3.5. In Vitro Testing of Cement Extracts, Live/Dead Staining of Cells, Gene Expression and Western Blotting

**Figure 8:**
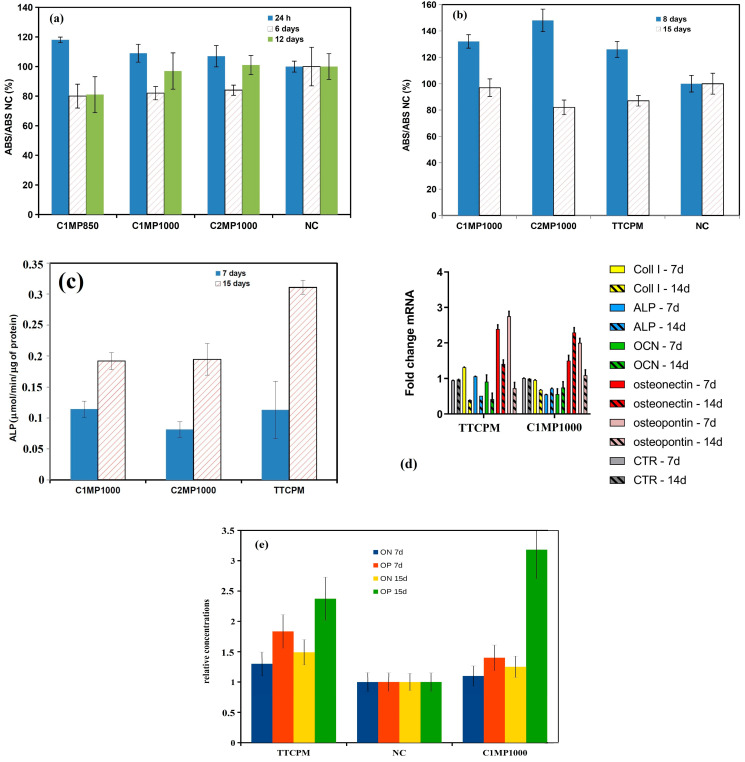
Viability of MSCߣs cultured in C1MP850, C1MP1000, and C2MP1000 extracts for 24 h, 6 days, 12 days (**a**); cement extracts during the prolonged cultivation period of up to 15 days (**b**); ALP activity of MSC´s cultured in TTCPM, C1MP1000, and C2MP1000 extracts (**c**) relative gene expression of COL1, OC, ON, OP, and ALP in MSCs cultured for 7 and 14 days in TTCPM and C1MP1000 extracts (**d**) and relative concentration of On and OP determined by the Western blot (SDS PAGE) analysis of lysates from MSCs cultured in 50% TTCPM and C1MP1000 extracts (**e**).

The evaluation of in vitro extract 24 h cytotoxicity according to ISO 10993-5:2009 is shown in Figure 8a. The relative viability of MC3T3E1 cells after 24 h of cultivation in magnesium cements extracts achieved 100–120% viability of cells in the negative control (NC) and reduction in relative viability down to around 80% of NC was found after 6 days of cultivation in osteogenic medium. Moreover, the relative viability of cells cultured in C1MP1000 and C2MP1000 rose to 100% of NC, in contrast to C1MP850, where no increase in proliferation activity was recorded after 12 days of culture. In the case of MSC’s cultured for a long time period in 50% cement extracts (Figure 8b), an enhanced cell proliferation was observed after 8 days of culture in all extracts other than NC. On the other hand, after 15 days of cultivation in extracts, a significant decrease in MSC´s viability was revealed as compared to NC. Note that the results confirmed the noncytotoxicity of cement extracts because relative viabilities of cells did not fall below the 70% limit defined in the standard. The rise in measured values of ALP activity of MSCs cultured in 50% cement extracts with time was demonstrated, and ALP activity cells in TTCPM extract was about 50% higher after 15 days of cultivation than in C1MP1000 or C2MP1000 extracts (Figure 8c).

From the comparison of analysis from RT PCR gene expression (Figure 8d) resulted a statistically significant upregulation of ON (osteonectin) and OP (osteopontin) genes in MSCs cultured in TTCPM and C1MP1000 extracts (*p* < 0.001), while ALP, COL1, and OC genes were downregulated in relation to control. The Western blot analysis of relative amounts of ON and OP secreted by MSCs during 7 and 15 days of cultivation in 50% TTCPM and C1MP1000 extracts confirmed enhanced secretion of these markers compared to NC (Figure 8e).

**Figure 9:**
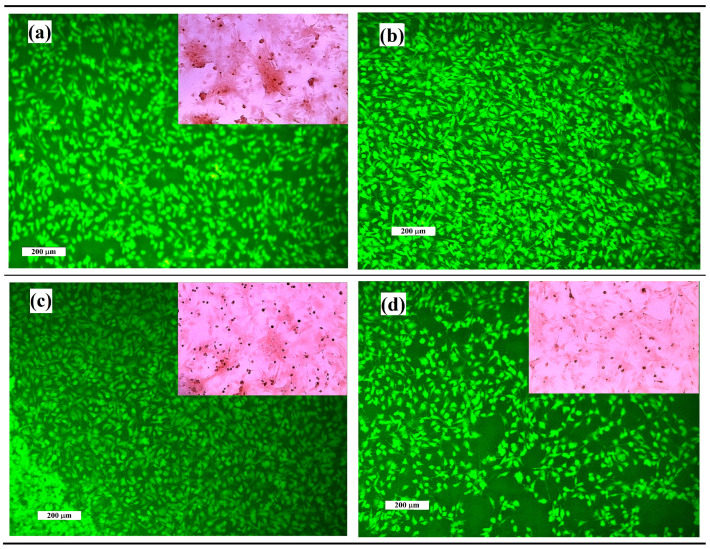
Morphology and distribution of osteoblast cells cultured on TTCPM (**a**), C1MP850 (**b**), C1MP1000 (**c**), and C2MP1000 (**d**) cements for 48 h (live/dead staining) and production of calcium deposits by MSCs cultured in 50% cement extracts for 15 days in details (Alizarin red staining).

The live/dead staining of osteoblasts cultured for 2 days on cement surfaces demonstrates the noncytotoxic character of cement surfaces and excellent adherence of cells to the surface of TTCPM and C1MP850 or C1MP1000 cements. The cells growing on Mg cement surfaces were well spread, well adhered, and uniformly distributed on surfaces of cements. The cells have a spindle-like prolonged morphology with longer filopodia mutually interconnected with adjacent cells. A lower cell density was observed on the C2MP1000 cement surface despite good spreading of viable cells, but no dead cells were found on the surface (Figure 9a–c). Figure 9d depicted the production of calcium deposits by MSCs cultured in 50% cement extracts and clearly show the higher content of deposits in TTCPM than C1MP1000 or C2MP1000 extracts.

## 4. Discussion

Magnesium phosphate cements are mostly based on a mixture of MgO with solutions of ammonium dihydrogen phosphate, potassium, or dihydrogen phosphates, and achieved a relatively high compressive strength of around 60 MPa after setting with various setting times from 12 to 17 min depending on the composition. In contrast to these suitable parameters for medical use, a major disadvantage of MPC is in the elution of heat from strong exothermic reactions during setting, with up to a 90 °C rise in temperature, which disqualified these cement types for biomedical applications [47] and supports the idea of finding other suitable Mg compounds to replace MgO. The bone contains various elements but the main inorganic impurities are sodium, chloride, magnesium, and carbonates [28]. In biological apatites, the amount of Mg increases with the degree of bone calcification, but the Mg content had little effect on changes in HAP lattice parameters as opposed to a decrease in their thermal stability. In an addition, the crystalite size and crystallinity of HAP increased with ageing from poorly crystalline, almost amorphous to nanocrystalline in older age, simultaneously with reduction in Mg content [3]. The low concentrations of Mg ions induced macrophages to the production of an anti-inflammatory cytokines and stimulated expression of BMP-2, then activated the BMP-2 signaling pathway in MSCs. It was shown that modification of conditioned media with 100 mg/L Mg^2^ ions caused a rise in the ALP activity of cells and upregulation of osteogenic genes (Runx-2, ALP, OP, and OC) [48]. Thus, the addition of magnesium to biocement mixtures can play a positive role for the activation of bone cells to resorption, remodeling, and new bone tissue formation during the healing of bone defects. It was found that the pyrophosphate ions suppressed the crystal growth of the calcium phosphate phase (composition close to octacalcium phosphate) on HAP microparticles, and pyrophosphates were mostly adsorbed on their surfaces. Moreover, the 15% of total pyrophosphate amount was hydrolyzed on the surface of HAP particles at pH = 7.4 [9]. Amorphous calcium pyrophosphates are often formed during the conversion of calcium phosphate-based cements with a pyrophosphate component in a solid powder mixture or in a liquid component added during the mixing of cement pastes. Amorphous calcium diphosphates were found in the surface coating of the formed brushite particles after transformation and hardening of a powder mixture of βTCP/Na_2_H_2_P_2_O_7_ powder mixture and 0.5 M citric acid as a liquid component [49]. The question is the stability of these types of amorphous compounds under physiological conditions, but the synthesis of amorphous calcium pyrophosphate has been demonstrated under physiological conditions even at higher temperatures or acidic pH from soluble calcium salt and pyrophosphate [50], which clearly confirms the possibility of the formation of similar amorphous calcium pyrophosphates in prepared cements. The strong refinement of HAP particles was the result of enhanced concentration of Mg ions, which affected the nucleation, growth, and dissolution of precipitated HAP particles during cement hardening in SBF, where especially increasing the solubility of HAP can cause high supersaturation of the local environment relative to HAP. The Mg substitution for Ca^2+^ site in the HAP lattice was preferred in Mg-doped carbonated HAP (up to 15 mol%) synthesized by mechanical alloying and clear amorphization, particle size reduction, and decreasing a, c-lattice parameters with increasing Mg content in the HAP lattice were found [51].

Similarly, a reduction in size, change in morphology to thin and needle-like, and the rise in solubility of Mg-doped HAP (5.7 mol%) nanoparticles prepared by the neutralization precipitation [52] were shown. Moreover, the solid titration method identified enhanced solubility of HAP in the presence of Mg ions in Hank´s solution even in mmolar concentrations [53]. The study of the effect Mg in the form of MgCl_2_ or Mg_3_(PO_4_)_2_ solutions in concentrations of Mg ions from 1 to 10 mM on the setting process of TTCP/DCPA cement systems showed a strong effect of Mg ions on the nucleation of calcium deficient HAP particles under 4 mM concentration while nucleation and growth were affected by further increasing the Mg concentration in hardening liquid. Besides, a stronger influence of soluble magnesium phosphate complexes on HAP nucleation was revealed as compared with MgCl_2_ solutions due to enhanced adsorption on the surface of HAP precipitates [10]. This influence was very active in TTCPM cement with the addition of dolomite or newberyite where the concentration of Mg^2+^ ions during the soaking of cement in SBF achieved more than fourfold values compared to those achieved with C2MP1000, and the local concentration exceeded 4 mM in the cements. One of the crucial aspects for the formation of magnesium-containing secondary phases in the HAP precipitation process, regardless of the synthesis conditions, is to know the solubility limit of Mg ions in the HAP lattice. In the case of 1 and 2 wt%, the addition of Mg to TTCPM cements, the total Mg^2+^ substitution for Ca^2+^ represented ~4 and 8 mol% relative to Ca^2+^. In Mg-doped HAP (18 mol%), inhibition of growth of precipitated particles with a decrease in the degree of crystallinity was demonstrated, as well as unchanged thermal stability of the HAP lattice, but a small amount of unsubstituted Mg in the form of Mg(OH)_2_ [28] was found, which suggests the solubility limit was exceeded. In contrast, no secondary phases were identified in Mg-doped HAP nanoparticles (10 mol% of Mg) prepared by aqueous precipitation [54]. Thus, the total content of Mg in C1MP and C2MP cements did not exceed the measured solubility of Mg close to 10 mol% of Mg in HAP lattice (which is consistent with the results of XRD analysis where no additional phases were identified), but in a local environment during setting both the solubility limit of Mg_2_P_2_O_7_ in aqueous liquid cement component and Mg ions in the HAP lattice could be achieved. These effects can cause a reduction in Mg_2_P_2_O_7_ dissolution as well as the formation of a relative compact surface coating, hindering further dissolution or transformation of the pyrophosphate particles.

The results of XRD and FTIR analysis clearly verified the almost complete transformation of Mg TTCPM cement mixture to nanocrystalline calcium deficient HAP after 7 days hardening in SBF, but residues of the Mg_2_P_2_O_7_ microcrystalline component were still present in the final cement. Note that none of the additional secondary magnesium-containing phases created from decomposed Mg_2_P_2_O_7_ were observed in the hardened cements. The comparison of XRD integral intensities after the fitting of patterns arose from reflection of a (012) plane of Mg_2_P_2_O_7_ in the origin powder mixtures (the most intense line) and hardened samples, which showed about 42% and 77% pyrophosphate conversion in C2MP1000 and C1MP1000 cements, respectively, which clearly demonstrates the slower solubility and almost the same amount of decomposed Mg_2_P_2_O_7_ (~0.5 wt% of Mg) in SBF regardless of its content in the cement. On the other hand, 29% of the original Mg_2_P_2_O_7_ was measured in the C1MP850 sample after setting in SBF, which could be the result of the lower crystallinity size of pyrophosphate in the sample (51 nm vs. 136 nm in Mg_2_P_2_O_7_ annealed at 850 and 1000 °C, respectively, calculated from the Scherrer´s equation and line from reflection of the (012) plane). A very small rise in concentration of Ca and Mg ions (<0.1 mM) in the SBF solution was found only during soaking of the C2MP1000 sample, and no difference was identified in C1MP cements, which clearly confirmed the consumption and substitution of Mg ions in the lattice of the newly formed hydroxyapatite phase, including phosphates after the dissolution and hydrolysis of pyrophosphates. Compared to TTCPM cement, where a rapid reduction in calcium and phosphate ion concentrations in SBF was measured during the first 6 h of soaking, only a slow decrease in SBF concentrations was observed with C1MP and C2MP samples. Besides the pH of the solution being insignificantly shifted to the basic region during soaking in SBF, resulting in an appropriate cement composition in relation to the setting process and the transformation of cement components. Contrary to the above facts, in the case C1MP or C2MP cements, a reduction in intensity of pyrophosphate peaks in the FTIR spectra of hardened cements more likely revealed their hydrolysis during soaking than a high degree of possible surface adsorption of pyrophosphates on the Hap particles, or Mg_2_P_2_O_7_ surface interaction with calcium ions, but these phases are difficulty identified due to the formation of very thin surface coatings.

Microstructure analysis of hardened C1MP1000 and C2MP1000 cements showed a reduction in HAP particle size with predominant spherical shaped nanoparticles joined to form agglomerates weakly mutually bound, or bound to the cement matrix as compared to TTCPM cement, which consisted of a large portion of longer needle-like HAP nanoparticles. The microstructure of C1MP1000 and C2MP1000 cement was not reinforced by the remains of coarser Mg_2_P_2_O_7_ particles identified in the microstructures despite the close contact with the surrounding very fine cement calcium phosphate matrix. This indicated the low strength of magnesium pyrophosphate particles, which are poorly sintered agglomerates of submicron nanoparticles, as well as the very fine precipitated spherical calcium phosphate particles nucleated on Mg_2_P_2_O_7_ surfaces weakly interconnecting the origin pyrophosphate with the surrounding cement matrix.

The CS of C1MP or C2MP cements (~14 MPa) was significantly reduced compared to TTCPM cement, which correlates with observed changes in the morphology of particles and agglomerates of the HAP phase in cement microstructures characterized by very fine spherical nanoparticles, but CS values are close to the trabecular bone. Note that the addition of the Mg_2_P_2_O_7_ phase had no influence on the ST of cements, as the slow dissolution of the phase allows continuous and gradual consumption of released Mg and phosphate ions during the cement transformation with substitution to the HAP lattice. In the case of cements with a high content of Mg phases, the one spot method synthesized whitlockite/stanfieldite/MgO cement powders with different [Ca + Mg]/[P] mole ratios (1.5–2), and 50% NaH_2_PO_4_ solution as a liquid component had a pH value close to physiological (~7.6), CS up to 22 MPa, ST around 10–15 min, and low cytotoxicity at concentrations up to 8 mM of Mg ions released from the cements [55]. Struvite magnesium phosphate cement with the addition of 10 wt.% Bi_2_O_3_ was studied for endodontic application and showed good sealing ability, mechanical properties (17–34 MPa), and setting time [56], but C1MP cement is not suitable for this use due to its poor mechanical strength and too high porosity (~50%). It was demonstrated that the combination of TTCPA/DCPA cement with a large fraction of Mg cement powder to the composite mixture improved the CS of final struvite/HAP cements (up to 91 MPa) while maintaining a short ST~6 min due to its compact microstructure formation and demonstrated excellent biodegradability with faster new bone ingrowth during the healing of artificial bone defects after their implantation into rabbit femurs [23]. The CS of cements rose with Mg_3_(PO_4_)_2_ content from 34 to 65 MPa for pure CPC and a cement mixture containing 20 wt% of Mg, respectively [25]. In a similar type of cement with a high ratio (1:1) of MPC and TTCP/DCPA, the transformation to HAP after setting in SBF with plate-like morphology of HAP particles and dissolution of Mg phase, almost double CS (~72 MPa) and a ST shortened to 6 min were confirmed [22].

In terms of the biological characteristics of cements, as in the case of unmodified calcium phosphate cements, a clear influence of microstructure features on cell attachment, proliferation, ALP activity in vitro, and new bone tissue growth after implantation were revealed and microporous/macroporous scaffolds prepared by NaCl leaching from TTCP/DCPA/struvite samples showed more favorable properties than macro- or micro-porous systems [24]. C1MP or C2MP cements had a relative dense microstructure with a large fraction of micropores with dimensions <10 µm, but the observed refinement of calcium phosphate particles in cements could effectively contribute to their resorption and enhancing of osteoblast activity during healing. It was demonstrated that the brushite/α-TCP/calcium pyrophosphate cement (up to 28%) showed significantly higher resorbability and increased new bone tissue formation than orthophosphate cement, probably as a result of the formation of amorphous HAP particles after enzymatic hydrolysis of pyrophosphate with ALP produced by active osteoblasts after the implantation to a bone defect [57]. In addition, increasing the activity of osteoclasts with a concentration of Mg^2+^ ions in medium (up to 10 mM) could positively affect the bioresorption of magnesium cements in vivo [26]. Gene expression analysis and Western blotting of MSCs cultured in C1MP1000 cement extracts verified the overexpression of OP and ON genes after 7 and 15 days of culture in osteogenic medium, as well as an increase in extracellular concentration of ON and OP after 15 days of culture. It is known that OP promotes osteoclasts anchoring to the mineral matrix of bones, which could be a suitable characteristic for the first stage of bone cement resorption [58] and enhanced levels of interfacial OP contribute to matrix mineralization and the proper integration of new bone tissue to mature bone [59]. In addition, osteonectin is expressed at high levels in active osteoblasts and supports bone mineral binding to collagens [60]. The correlation between Mg content in TTCP/DCPA cement modified by the addition of 5–20 wt% of MgO/Ca(H_2_PO_4_)_2_.H_2_O powder mixture on cell adherence and gene expression clearly demonstrated the direct effect of cements surfaces on MSCs activity after seeding and cultivation, where cells cultured on Mg cement containing 5 wt% Mg mixture showed enhanced adsorption of fibronectin, better cell spreading on surfaces, and over expression of collagen I, OC, and ALP osteogenic gene markers. The higher extracellular concentration of Ca^2+^ and Mg^2+^ ions (~2.5 mM) had an insignificant effect on MSCs differentiation, suggesting that cellular activity probably resulted from direct contact of cells with insoluble Mg compounds (microcrystalline Mg_3_(PO_4_)_2_) in cements [25]. Thus, a stronger effect of magnesium pyrophosphate on cellular activity could be observed after seeding of cells on cement surfaces, but on the other hand, this effect needs to be thoroughly analyzed as the accuracy of the results can be affected by incorrect surface structure. Numerical simulation to predict cellular processes verified experimental data on the stimulatory effect of Mg at 3–6 mM concentration on proliferation and ALP activity as well as OC inhibition as a late differentiation marker of MSCs [61], but this level was not reached in C1MP or C2MP cement extracts; however, the local ion concentration may be higher in close contact between cements and cells or tissues. Magnesium (3 mM) was found to be able to effectively reduce β-glycerol phosphate-induced calcification in rat vascular smooth muscle cells by regulating ALP and OP gene expression [62]. For C1MP1000 cement, the ALP activity of MSCs rose with culture time, but small amounts of calcium deposits produced by cells after 15 days of culture, as well as low expression of ALP and OC genes may indicate that MSCs were not fully differentiated to mature osteoblasts. It is clear from the above facts that Mg ions have a complex effect on bone cell activity and the choice of magnesium compound to modify the TTCPM cement mixture must take into account the physico-chemical properties of the final cement mixture. In the future, it will be interesting to focus research on magnesium-doped calcium phosphate phases suitable as cement components or to evaluate other magnesium or potassium-containing magnesium phosphates that could be degraded more rapidly in CPC with a slower release of magnesium in the first setting stages.

## 5. Conclusions

Magnesium modified TTCPM cement mixtures with 1 and 2 wt% of magnesium were prepared by mixing powdered magnesium pyrophosphate and TTCPM in a ball mill. Complete conversion of the calcium phosphate cement component with residues of the original Mg_2_P_2_O_7_ phase was found after 7 days of cement soaking in SBF. Moreover, the same amounts of Mg_2_P_2_O_7_ phase were decomposed regardless of total addition to the cement. Only small increases in the pH and the concentration of magnesium ions in the SBF during the soaking of the cements were measured, which confirm their continuous and gradual consumption into the HAP lattice during setting, with insignificant effects on the setting process and setting time of magnesium modified cements. The cements had CS of ~14 MPa and short setting times of 5–10 min. In magnesium modified cements, refinement of the cement microstructure was observed with the formation of spherical HAP particles joined to the form of spherical agglomerates weakly bound to each other. No cytotoxicity of cements and their extracts was detected and increased proliferative activity of cells was identified as well as up-regulation of OP and ON genes in MSCs cultured for 7 and 15 days in cement extracts.

## Data Availability

Data sharing is not applicable.
